# Theoretical Study of CO, NO, NO_2_, Cl_2_, and H_2_S Adsorption Interactions with PdO–Graphene Composites for Gas Sensor Applications

**DOI:** 10.3390/mi16010009

**Published:** 2024-12-25

**Authors:** Piumantha Samaranayake, Azeez Ahamed, Visal de Silva, Nadeesha Manohari Wickramage, Muhammad Raziq Rahimi Kooh, Roshan Thotagamuge

**Affiliations:** 1 Department of Physics, Faculty of Science, University of Ruhuna, Matara 81000, Sri Lanka; samaranayakepiumantha@gmail.com (P.S.); azeezahamed1997@gmail.com (A.A.); desilvavisal@gmail.com (V.d.S.); 2 Centre for Advanced Material and Energy Sciences, Universiti Brunei Darussalam, Jalan Tungku Link, Gadong BE 1410, Brunei; raziq.kooh@ubd.edu.bn; 3 Department of Optometry, Faculty of Allied Health Sciences, University of Sri Jayewardenepura, Nugegoda 10250, Sri Lanka

**Keywords:** PdO–graphene composite, density functional theory, gas sensor sensitivity, adsorption energy, charge transfer

## Abstract

Gas sensors play a vital role in detecting gases in the air, converting their concentrations into electrical signals for industrial, environmental, and safety applications. This study used density functional theory methods to explore the mechanism and sensitivity of a PdO–graphene composite sensor towards various gases (CO, NO, NO_2_, H_2_S, and Cl_2_). All calculations, including structure, energy, and frequency optimizations, were performed using the Gaussian software with appropriate configurations and basis sets. Key parameters such as the adsorption energy, charge transfer, energy gap, density of states, and HOMO–LUMO were computed for each gas molecule on the PdO–graphene composite. The sensitivity and recovery time were also evaluated. The findings show that CO exhibited the highest adsorption energy (−6.5513 eV) and adsorbed with a noticeable tilt toward the PdO–graphene plane, indicating a strong interaction, and H_2_S exhibited the lowest adsorption energy, calculated as −2.0110 eV. H_2_S demonstrated the highest charge transfer of 0.445 e and an energy gap of 3.1321 eV, and CO exhibited the lowest charge transfer, calculated as 0.036 e, while NO_2_ demonstrated the lowest energy gap, determined to be 2.5004 eV. NO_2_ demonstrated the highest sensitivity, at 1285.2% for the PdO–graphene composite, and the lowest were Cl_2_ and H_2_S, with a sensitivity of 99.9%, while Cl_2_ had the shortest recovery time of 7.66 × 10^−11^ s, and CO had the longest recovery time of 2.55 × 10^−10^ s. The addition of PdO significantly enhanced the interaction strength between the adsorbed gas molecules and the graphene sheet when compared to Pd–graphene or pure graphene. This enhancement is reflected in the increased adsorption energy and band gap and low charge transfer, which significantly influenced the electrical conductivity of the PdO–graphene sheet. In conclusion, the incorporation of PdO into graphene improves the sensitivity of the gas sensor, particularly for detecting NO_2_, making PdO–graphene a highly suitable material for gas sensing applications.

## 1. Introduction

The development of highly sensitive sensors for detecting dangerous gases has garnered significant attention in recent years due to their critical importance across various industries and applications. The air we breathe contains a variety of gases, some of which pose serious health risks or act as atmospheric pollutants, while others are integral to industrial and medicinal processes. Given that humans, plants, and animals are the primary inhabitants of our ecosystem, it is paramount to identify the presence of these gases to ensure safety.

Among the harmful gases, carbon monoxide (CO) and nitric oxide (NO) have particularly detrimental impacts on human health and lifestyle. CO is a poisonous, tasteless, colorless, and odorless gas that can cause symptoms such as headaches and dizziness at low concentrations (approximately 35 ppm) and can be fatal at higher concentrations (above 150 ppm) [1,2]. NO, though widely used in industrial and environmental applications, is highly toxic and requires close monitoring. Hydrogen sulfide (H_2_S), another hazardous gas, is commonly released from industrial processes such as coking plants, wastewater treatment units, natural gas and biogas processing, and petroleum refineries [3]. Exposure to H_2_S is extremely hazardous and causes serious health effects, ranging from mild irritation, such as a runny nose and coughing, to coma and death. Additionally, it can corrode pipelines and process equipment, impacting industrial operations [4]. Similarly, nitrogen dioxide (NO_2_), produced by fuel combustion, biomass burning, and certain microbial processes in agriculture, is known to cause respiratory inflammation, reduced lung function, increased risk of respiratory disorders, and heightened sensitivity to allergens [5]. Chlorine (Cl_2_), despite its industrial uses, is a toxic greenish-yellow gas with an occupational exposure limit (OEL) of 0.5 ppm, capable of causing skin irritation, suffocation, sensory irritation, bronchospasm, and other severe symptoms.

Numerous materials have been studied for hazardous gas adsorption, including metal oxides, ionic liquids, zeolites, and carbon-based materials. The unique properties of low-dimensional nanostructures, such as carbon nanotubes, fullerenes, pristine graphene, metal oxides, decorated graphene, and doped graphene, have opened new research avenues for gas molecule adsorption [6,7]. Research interest in graphene and related two-dimensional materials has grown significantly [8]. Their unique structure allows for enhanced interaction with gas molecules, enabling high sensitivity and selectivity in detecting various gases. For example, among the 2D materials, MXenes stand out promising due to their extraordinary electrical conductivity, chemical tenability, and mechanical robustness. MXenes offer a combination of high conductivity, large specific surface area, and tuneable surface functional groups, making them highly effective in creating gas sensors with rapid response and recovery times [9]. Graphene and its derivatives, such as reduced graphene oxide (RGO), pristine graphene (PG), and graphene oxide (GO), have emerged as promising candidates due to their exceptional properties, including their large surface area, low electrical noise, high mechanical strength, good thermal stability, and high carrier mobility at room temperature [10,11,12]. Graphene’s high gas adsorption capability has made it a promising candidate for gas sensing applications, capable of detecting single molecules [13]. However, the interaction between pure graphene and gas molecules is relatively weak, characterized by low adsorption energy and minimal charge transfer, limiting the sensitivity of graphene-based sensors [14,15,16,17].

Recent theoretical studies have explored the use of graphene-embedded devices as superior catalysts for gas molecule oxidation/reduction reactions [18,19,20]. The incorporation of palladium (Pd) dopants into graphene can induce local surface curvature, alter the electronic structure, and enhance gas sensitivity [21]. Pd-doped graphene (Pd-G) has demonstrated improved sensitivity to various gases, including CO, NH_3_, O_2_, and NO_2_, compared to pure graphene [21]; specifically, the induced surface curvature breaks the symmetry of the graphene lattice, creating localized regions of higher chemical reactivity. This, in combination with the altered electronic density near the Fermi level, plays a crucial role in enhancing the material’s interaction with gas molecules. The introduction of Pd atoms introduces additional electronic states near the Fermi level, which directly affect the distribution of electrons around the Pd sites. These states act as active centers for gas adsorption, allowing the material to interact more strongly with incoming gas molecules. However, pure Pd is expensive and vulnerable to sulfur contamination, which deactivates its catalytic sites. Palladium oxide (PdO) is a more stable alternative, offering better resistance to sulfur contamination, especially in high-temperature or harsh environments. PdO maintains its catalytic activity over time by preventing sulfur from binding to its surface.

**Figure 1:**
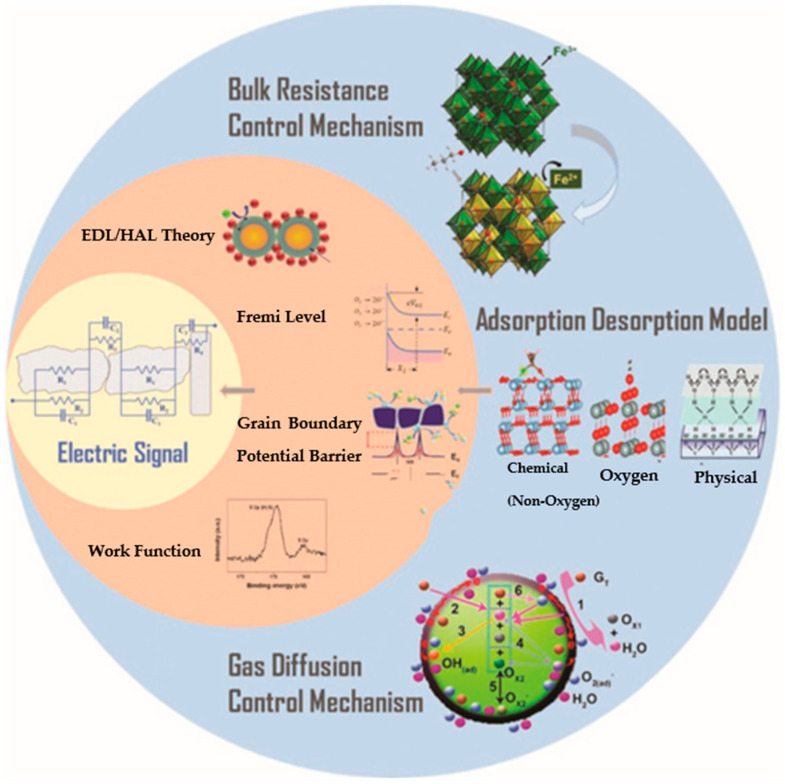
Gas sensing mechanism of MOS.

In metal oxide semiconductor gas sensors (MOS), PdO enhances both sensitivity and selectivity, particularly for hydrogen detection. It provides additional active sites for hydrogen adsorption and dissociation, leading to greater sensitivity. When combined with metal–organic frameworks (MOFs), the combination leverages the large surface area of MOFs for gas adsorption, while PdO enhances the selectivity, allowing for accurate hydrogen detection with minimal interference from other gases. This synergy results in a more stable, durable, and efficient gas sensing system [22,23,24,25]. The primary reason that common MOS gas sensors can identify a gas is due to the change in the electrical signal caused by the gas. The ability of a gas to alter a sensor’s electrical characteristics is explained by gas sensing processes. There are two categories into which the commonly utilized gas sensing mechanisms fall as in Figure 1. One category, which includes mechanisms such as Fermi-level control theory, grain boundary barrier control theory, and EDL (electron depletion layer)/HAL (hole accumulation layer) theory, explains the changes in electrical characteristics from a comparatively small perspective.

Changes in electrical characteristics in any application inevitably lead to alterations in physical characteristics such as energy bands and work functions. These ideas are more theoretical. The interaction between materials and gases is the primary topic of the other, more macroscopic, theory category. This type of theory includes the adsorption/desorption model, the bulk resistance control mechanism, and the gas diffusion control mechanism [26].

This study employs density functional theory (DFT) computations to investigate the structural, electronic, and catalytic properties of PdO-doped graphene (PdO-G) composites. The objective is to demonstrate that PdO-G can offer superior sensitivity and selectivity for gas sensing compared to pure graphene and Pd-G. This research aims to advance the development of gas sensors capable of detecting hazardous gases with high precision and reliability, enhancing safety in various industrial and environmental settings.

## 2. Computational Methodology

In this study, all calculations were conducted employing the unrestricted DFT method due to its established accuracy in characterizing structural, electrical, and magnetic properties. Specifically, Becke’s three-parameter hybrid exchange-correlation functional, B3LYP, coupled with the Los Alamos National Laboratory 2 Double-Zeta (LANL2DZ) basis set, as implemented in the Gaussian 16 software package, was chosen for the computational simulations [27,28]. The LANL2DZ basis set is tailored for transition-metal-containing systems, comprising a larger basis set for valence electrons and a relatively smaller one for inner core electrons.

The selection of the B3LYP/LANL2DZ level of theory [29,30] is commonly recognized as a favorable compromise for systems involving transition metals, balancing computational efficiency with accuracy. To prevent structural distortions, the graphene surface model consisted of 24 carbon atoms and 12 terminal hydrogen atoms [31]. Geometry optimizations were performed based on the typical hybrid exchange-correlation function of B3LYP, considering the ground-state energy level with a single-spin multiplicity and net neutral charge.

The modeling of the lowest unoccupied molecular orbital (LUMO) and the highest occupied molecular orbital (HOMO) with respect to the vacuum level was carried out, along with a Mulliken charge population analysis. The Total Density of States (TDOS) and non-covalent interactions (NCIs) were analyzed and visualized using the Multiwfn v3.8 software package [32].

## 3. Results and Discussion

DFT calculations were employed to design graphene-based gas sensors for detecting CO, NO, NO_2_, Cl_2_, and H_2_S. A novel PdO-G composite was developed by doping graphene with Pd and O atoms to enhance its gas sensing performance.

**Figure 2:**
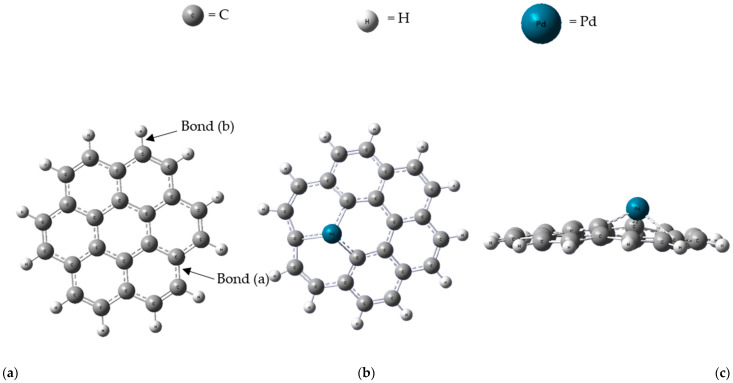
(**a**) Optimized graphene sheet and (**b**) top view and (**c**) side view of optimized Pd-G.

The initial investigation examined the geometry of Pd-G for gas adsorption. Substituting a Pd atom for a C atom in the graphene sheet resulted in a notable structural distortion, as depicted in Figure 2b,c. This substitution caused deformation in the six-membered ring (6MR) adjacent to the doping site, alleviating stress and causing the Pd atom to protrude from the graphene sheet [21]. This structural modification plays a critical role in enhancing gas adsorption.

**Figure 3:**
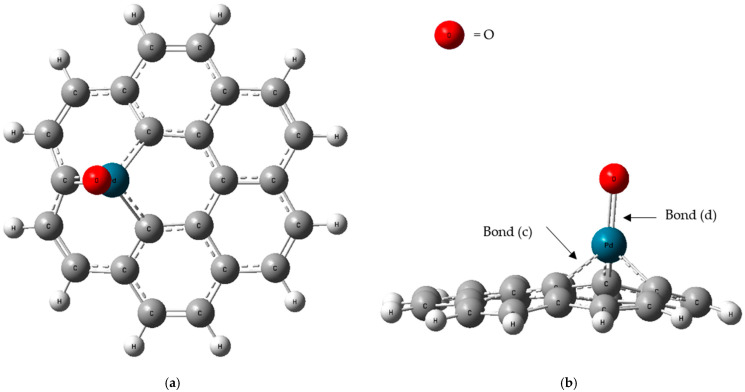
(**a**) Top and (**b**) side view of optimized PdO-G.

The optimization process focused on obtaining the most thermodynamically stable configuration, and the optimized structure of the PdO-G composite is illustrated in Figure 3a,b.

Graphene optimization was conducted before being incorporated into a composite with PdO clusters. The composite formation involved the substitution of a C atom in the graphene lattice with a Pd atom, followed by the attachment of an O atom to the Pd atom. This process was optimized at the ground-state energy level. Figure 2 illustrates the ground-state optimized structures of both pristine graphene and the PdO-G composite. The average bond lengths of carbon–carbon (C-C) (Bond (a)) and carbon–hydrogen (C-H) (Bond (b)) bonds in the optimized graphene sheet were determined to be 1.434 Å and 1.087 Å, respectively. Figure 3 shows the top and side views of the optimized PdO-G composite structure.

Upon doping a Pd atom into graphene, it forms bonds with the three nearest C atoms. The graphene surface undergoes slight distortion, causing the Pd atom to protrude from the graphene plane. This distortion is attributed to the larger atomic radius of Pd compared to that of C [33]. The average bond lengths between palladium and carbon (Pd-C) (Bond (c)) and between palladium and oxygen (Pd-O) (Bond (d)) bonds were measured to be 1.964 Å and 1.808 Å, respectively.

**Figure 4:**
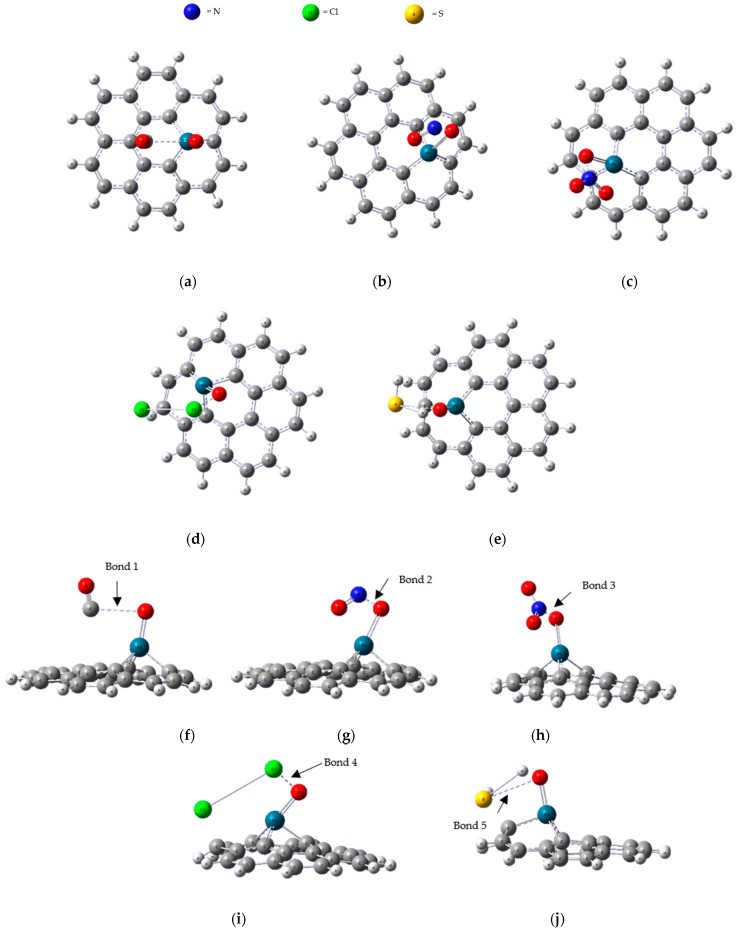
Top views of PdO-G composite after adsorption of (**a**) CO, (**b**) NO, (**c**) NO_2_, (**d**) Cl_2_, and (**e**) H_2_S, along with side views of subsequent adsorptions of (**f**) CO, (**g**) NO, (**h**) NO_2_, (**i**) Cl_2_, and (**j**) H_2_S.

Figure 4 depicts the most optimal adsorption structures of CO, NO, NO_2_, Cl_2_ and H_2_S adsorbed onto the PdO-G composite following complete relaxation. Table 1 provides the calculated values of adsorption energy (E_ads_), charge transfer (Q_mulliken_), HOMO energy (E_HOMO_), LUMO energy (E_LUMO_), and band gap (E_g_) for each adsorption system. 

### 3.1. CO Adsorption

The CO gas molecule was adsorbed obliquely (Bond 1) onto the PdO graphene composite, with the C atoms positioned closer to the O atom within the composite. The bond lengths between carbon and oxygen (C-O) elongated to 1.166 Å, while the CO molecule retained its linear structure. The adsorption energy for this adsorption configuration was calculated to be −6.5513 eV, which is higher than that observed for CO adsorption on PG (E_ad_ = −0.08 eV) [21]. The distance between the CO molecule and the substrate was measured at 2.779 Å, significantly shorter than the distance observed for CO adsorption on PG (d = 3.22 Å) [21], suggesting a chemisorption mechanism. The CO bond exhibited a notable tilt towards the plane of PdO-G, with the C atom in close proximity to the O atom. This configuration was accompanied by a substantial increase in adsorption energy and a notable reduction in adsorption length. These observations collectively suggest a heightened strength in the interaction between CO and PdO-G. The charge transfer, denoted as Q = 0.036 e, observed in CO adsorbed on PdO-G was an order of magnitude higher than the value of 0.015e observed in CO adsorbed on PG [21]. This significant disparity indicates a distinct alteration in the local electronic structure of PdO-G, thereby highlighting the sensitivity of CO to this structural variation. The band gap after the adsorption of a CO molecule on PdO-G was calculated to be 2.5905 eV. This value represents the energy difference between the highest HOMO and the LUMO of the adsorbate–substrate system. The observed band gap alteration indicates modifications in the electronic structure of PdO-G upon CO adsorption, potentially due to changes in the charge distribution and electronic states near the Fermi level. The investigation of the impact of PdO impurity on the reactivity of graphene towards CO molecules involved the computational determination of the TDOS for PdO-G with adsorbed CO molecules. This analysis was conducted to elucidate the electronic structure alterations induced by PdO impurity and CO molecule adsorption, thereby providing insights into the reactivity enhancement or inhibition mechanisms in graphene-based systems. Upon the analysis, it was observed that there was no discernible shift in the peak of the TDOS before and after the adsorption of CO molecules onto PdO-G. This observation suggests that introducing CO molecules does not significantly alter the electronic structure localized around the PdO impurity in the graphene matrix. The observed decrease in the energy gap following CO adsorption onto the PdO-G cluster implies heightened reactivity towards CO molecules. This reduction in the band gap signifies a lowering of the energy barrier for the adsorption reaction to occur, suggesting an enhanced affinity and interaction between CO molecules and the PdO-G cluster. Consequently, the system becomes more reactive towards CO, indicating a potential catalytic effect facilitated by the PdO impurity within the graphene matrix. This insight underscores the significance of the PdO impurity in modulating the reactivity of graphene-based materials towards CO molecules, with potential implications for various catalytic applications.

### 3.2. NO Adsorption

Figure 4b,g depict the fully relaxed structure of adsorbed NO gas molecules on PdO-G after geometrical optimization. Notably, the nitrogen (N) atom of the NO molecule was observed to be in close proximity to the O atom within the PdO moiety. This spatial arrangement suggests a favorable adsorption configuration, potentially indicating the formation of chemical bonds or strong adsorbate–substrate interactions between NO and the PdO-G surface. The adsorption energy for NO on PdO-G was calculated to be −4.7706 eV, with a distance of 1.320 Å between the graphene surface and the NO molecule. Additionally, a charge transfer of −0.096 e was observed from the graphene to the NO molecule. The negative adsorption energy value indicates that the adsorption process is exothermic, meaning that energy is released during the adsorption of NO on the PdO-G surface. This energetically favorable interaction suggests strong adsorbate–substrate interactions and supports the stability of the NO adsorption configuration on PdO-G. The larger adsorption energy of −4.7706 eV and the smaller bond length of 1.320 Å between the graphene surface and the NO molecule observed in the adsorption of NO on PdO-G compared to other systems [34] indicate strong and stable chemical adsorption (Bond 2). The higher adsorption energy suggests that more energy is released upon the adsorption of NO on PdO-G, indicating stronger binding between NO and the PdO-G surface. The smaller bond length indicates a shorter distance between the NO molecule and the PdO-G surface, further corroborating the strong adsorbate–substrate interaction. These characteristics highlight the enhanced stability and reactivity of the NO adsorption configuration on PdO-G. The band gap after the adsorption of a NO molecule on PdO-G was calculated to be 2.5508 eV. The TDOS of NO adsorbed on PdO-G exhibited significant alterations, particularly in the vicinity of the Fermi level. Notably, peaks in the TDOS spectrum were observed to undergo shifts compared to the PdO-G system. These shifts indicate modifications in the electronic structure induced by the adsorption of NO molecules on the PdO-G surface. The changes near the Fermi level are particularly noteworthy, as they reflect alterations in the electronic states close to the Fermi energy, which could have implications for the reactivity and electronic properties of the adsorbate–substrate system.

### 3.3. NO_2_ Adsorption

The adsorption of NO_2_ on PdO-G results in the formation of a distinct chemical configuration known as cycloaddition. In this configuration, the N atom of the NO_2_ molecule forms a chemical bond with an O atom present in the PdO impurity (Bond 3). Furthermore, an O atom from the NO_2_ molecule orients downward towards the graphene surface. This cycloaddition configuration highlights the formation of chemical bonds between the NO_2_ molecule and the PdO-G system, mediated by the interaction between the N atom and the O atom in PdO. The adsorption energy and adsorption length for NO_2_ on PdO-G were calculated to be −3.1665 eV and 1.345 Å, respectively, indicating a chemisorption process. This suggests that NO_2_ forms strong chemical bonds with the PdO-G substrate rather than weaker physical adsorption. Moreover, a charge transfer of −0.199 e was observed from the substrate to NO_2_, indicating an electron transfer from the PdO-G substrate to the NO_2_ molecule upon adsorption. This charge transfer signifies a modification of the electronic structure of the NO_2_ molecule due to its interaction with the PdO-G substrate, highlighting the influence of the substrate on the chemical properties of the adsorbate. The band gap after the adsorption of a NO_2_ molecule on PdO-G was calculated to be 2.5004 eV. The TDOS of PdO-G upon NO_2_ adsorption reveals significant alterations compared to PdO-G. Specifically, the TDOS exhibits upward shifts in related energy levels, indicating NO_2_ acting as an acceptor species. Notably, a prominent peak emerges near the Fermi level, primarily attributed to Pd-4d orbitals. Weak hybridization occurs between Pd-4d orbitals and the O-2p and N-2p orbitals of NO_2_ within this region. Consequently, PdO-G demonstrates the potential for detecting NO_2_ molecules due to the distinctive electronic structure modifications induced by NO_2_ adsorption, which can be exploited for sensor applications.

### 3.4. H_2_S Adsorption

In the adsorption scenario of H_2_S molecule on PdO-G, it was observed that H_2_S remains undecomposed and is arranged nearly parallel to the PdO-G composite surface. This arrangement suggests a physisorption mechanism, where H_2_S molecules are weakly bound to the PdO-G surface through van der Waals interactions (Bond 4) without undergoing significant chemical transformations. The nearly parallel orientation implies a favorable adsorption configuration, possibly influenced by the electronic structure and surface properties of the PdO-G composite. This can be attributed to the saturated electronic d shell (d^10^) of Pd. Despite this, H_2_S undergoes chemisorption on the PdO-G surface, as evidenced by the strong hybridization and distinct electron cloud accumulation region observed between the H_2_S molecule and Pd atom. This phenomenon suggests the formation of chemical bonds between H_2_S and the PdO-G substrate, likely facilitated by the interaction between the lone pairs of electrons in the sulfur atom of H_2_S and the d orbitals of the Pd atom. The calculated adsorption energy and adsorption length for H_2_S on PdO-G were found to be −2.0110 eV and 3.137 Å, respectively. This indicates a relatively strong adsorption interaction between H_2_S and the PdO-G surface. Furthermore, it was noted that one of the hydrogen atoms in the H_2_S molecule is positioned in close proximity to the O atom in the PdO-G structure. This spatial arrangement suggests a favorable adsorption configuration, potentially facilitating interactions between the H_2_S molecule and the PdO-G surface, which could involve the formation of chemical bonds or strong adsorbate–substrate interactions. The observed charge transfer in H_2_S adsorbed on PdO-G was calculated to be +0.445 e. This positive charge transfer indicates an electron transfer from the PdO-G substrate to the H_2_S molecule upon adsorption. Consequently, the adsorption of H_2_S on PdO-G results in a partial positive charge accumulation on the H_2_S molecule, suggesting a modification of the electronic structure of H_2_S due to its interaction with the PdO-G surface. The band gap after the adsorption of an H_2_S molecule on PdO-G was calculated to be 3.1321 eV. The TDOS of PdO-G upon H_2_S adsorption demonstrates notable alterations compared to the PdO-G state. Specifically, the TDOS displays upward shifts in related energy levels, indicative of changes in the electronic structure induced by H_2_S adsorption. Notably, a prominent peak emerges near the Fermi level, suggesting significant electronic interactions between H_2_S molecules and the PdO-G substrate. This peak near the Fermi level may arise from the hybridization of H_2_S molecular orbitals with the electronic states of the PdO-G surface.

### 3.5. Cl_2_ Adsorption

In the adsorption scenario of Cl_2_ molecule on PdO-G, it was observed that a chlorine atom forms a bond with the O atom in PdO-G. The bond length of the Cl_2_ molecule increases approximately two-fold after adsorption compared to its length before adsorption. This suggests a significant alteration in the Cl-Cl bond upon adsorption onto the PdO-G surface. The bonding between one Cl atom and the O atom in PdO-G (Bond 2) indicates chemisorption, where chemical bonds are formed between the adsorbate and the substrate. The elongation of the Cl_2_ molecule’s bond length further underscores the interaction between Cl_2_ and PdO-G, potentially due to the formation of chemical bonds or strong adsorbate–substrate interactions. The adsorption energy for Cl_2_ on PdO-G is was calculated to be -1.9674 eV, indicating a moderately strong adsorption interaction between Cl_2_ and the PdO-G surface. The distance between the graphene surface and the Cl_2_ molecule was found to be 1.854 Å, suggesting a relatively close proximity between the adsorbate and the substrate. A charge transfer of -0.07 e was observed from the graphene to the Cl_2_ molecule, indicating an electron transfer from the substrate to the adsorbate upon adsorption. These findings suggest that Cl_2_ adsorption on PdO-G involves chemisorption, characterized by the formation of chemical bonds between Cl_2_ and the PdO-G surface. The moderately strong adsorption energy, close proximity between the adsorbate and substrate, and observed charge transfer all contribute to the stability of the Cl_2_ adsorption configuration on PdO-G. The band gap after the adsorption of a Cl_2_ molecule on PdO-G was calculated to be 3.0344 eV. The TDOS of PdO-G upon Cl_2_ adsorption displays noticeable alterations compared to the PdO-G state. Similar to the pattern observed after the adsorption of H_2_S molecules on PdO-G, the TDOS exhibits upward shifts in related energy levels. This indicates significant modifications in the electronic structure induced by the adsorption of Cl_2_ molecules on the PdO-G surface. Notably, a prominent peak emerges near the Fermi level in the TDOS spectrum. This peak suggests the presence of electronic states associated with the interaction between Cl_2_ molecules and the PdO-G substrate near the Fermi level. Such alterations near the Fermi level are crucial, as they may influence the reactivity and electronic properties of the adsorbate–substrate system. The similarity in the alterations observed in the TDOS after Cl_2_ and H_2_S adsorption on PdO-G suggests common underlying mechanisms in the adsorption behavior of these molecules on the PdO-G surface.

To calculate the adsorption energy (E_ads_) of gas molecules on the surface of the PdO-G composite, the following equation is typically used [35,36,37]:

(1) $$E_{\mathrm{ads}}=E_{\mathrm{PdO}-G+\mathrm{gas}\;\mathrm{molecule}}-\left(E_{\mathrm{PdO}-G}+{\;E}_{\mathrm{gas}\;\mathrm{molecule}}\right)$$

where $E_{\mathrm{PdO}-G+\mathrm{gas}\;\mathrm{molecule}}$ is the total energy of the PdO-G composite with the adsorbed molecule, $E_{\mathrm{PdO}-G}$ is the total energy of the PdO-G composite, and $E_{\mathrm{gas}\;\mathrm{molecule}}$ is the total energy of the isolated gas molecule.

The adsorption energy is obtained by subtracting the total energy of the PdO-G composite and the isolated gas molecule from the total energy of the PdO-G composite with the adsorbed molecule. This value represents the energy change associated with the adsorption process and indicates the strength of the interaction between the gas molecule and the PdO-G composite surface. Typically, a negative adsorption energy indicates an exothermic adsorption process, meaning that energy is released when the gas molecule interacts with the surface. The calculated adsorption energy for the gas molecules’ interaction with graphene and its composites is tabulated in Table 1.

The adsorption energy values obtained for various gas molecules (CO, NO, NO_2_, Cl_2_, H_2_S) on the surface of the PdO-G composite exhibit a distinct trend: CO: −6.5513 eV, NO: −4.7706 eV, NO_2_: −3.1665 eV, Cl_2_: −1.9674 eV, and H_2_S: −2.0110 eV.

Comparing these results, it is evident that CO exhibits the highest adsorption energy, followed by NO, NO_2_, H_2_S, and Cl_2_. This trend suggests that CO has the strongest interaction with the PdO-G composite surface, while Cl_2_ exhibits the weakest interaction among the considered gas molecules. The variations in adsorption energy can be attributed to the differing chemical properties of the gas molecules and their interactions with the PdO-G surface. Factors such as the electronic structure, polarizability, and chemical bonding characteristics of the molecules play crucial roles in determining the strength of their adsorption on the surface. The high adsorption energy observed for CO can be explained by its strong affinity for metal surfaces and the potential formation of strong chemical bonds with Pd atoms. On the other hand, Cl_2_, being a diatomic molecule with relatively weaker bonding compared to CO, exhibits lower adsorption energy values.

**Figure 5:**
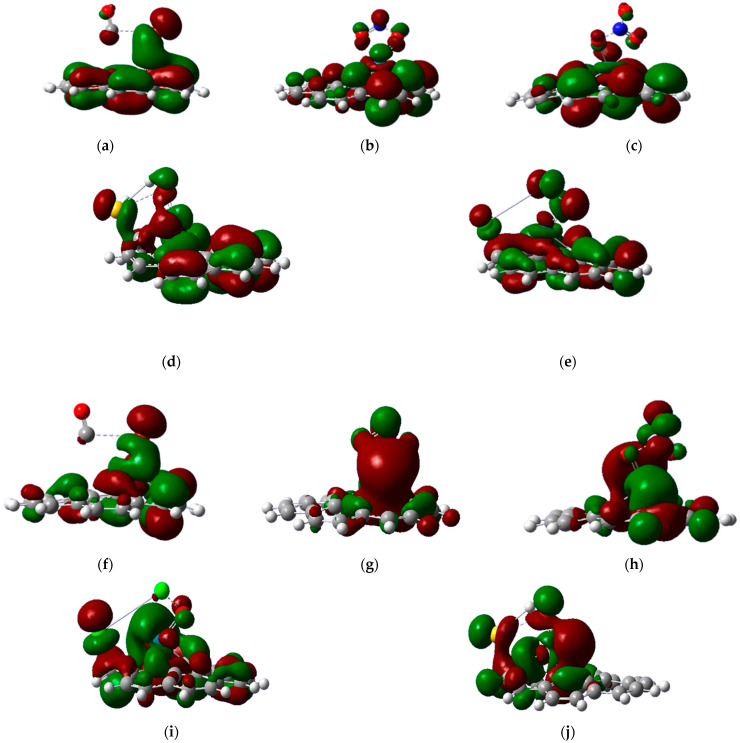
HOMO energy profiles of PdO-G after adsorption of (**a**) CO, (**b**) NO, (**c**) NO_2_, (**d**) Cl_2_, and (**e**) H_2_S, along with LUMO energy profiles after adsorption of (**f**) CO, (**g**) NO, (**h**) NO_2_, (**i**) Cl_2_, and (**j**) H_2_S, respectively.

The HOMO and LUMO energy profiles are shown in Figure 5 and the energy gap (E_g_) between the HOMO and LUMO energy levels was calculated as follows [38,39,40,41]:

(2) $$E_{g}={\;E}_{\mathrm{LUMO}}-{\;E\;}_{\mathrm{HOMO}}$$

where E_LUMO_ and E_HOMO_ are the energy values of LUMO and HOMO, respectively. The HOMO–LUMO energy gap was observed to increase significantly for both the Cl_2_ and H_2_S molecules upon adsorption on the PdO-G composite surface. This increase indicates a modification in the electronic structure of the adsorbed molecules, characterized by a larger energy difference between the highest occupied and lowest unoccupied molecular orbitals. The significant increase in the HOMO–LUMO energy gap suggests a stabilization of the electronic structure of the adsorbed molecules upon interaction with the PdO-G surface. This could be attributed to changes in the molecular geometry, electronic distribution, or intermolecular interactions induced by adsorption.

Mulliken’s charge population analyses were utilized to elucidate the charge transfer occurring within the system. This analysis involves calculating the charge transfer using the Mulliken charge analysis method, which is typically computed using the following equation [42,43,44]:

(3) $$\Delta Q={\;Q}_{i}-{\;Q\;}_{o}$$

where ΔQ is the charge transfer of the gas, and $Q_{i}$ and ${Q\;}_{o}$ are the Mulliken charges of the gas molecules after and prior to the adsorption. By subtracting the charge of the substrate from the charge of the adsorbate molecule, the Mulliken charge transfer is obtained. This value quantifies the degree of charge transfer between the adsorbate and the substrate, providing insights into the electronic interactions and bonding at the adsorbate–substrate interface.

As depicted in Table 1, the ΔQ Mulliken values exhibit an increasing trend from NO_2_ to H_2_S, where H_2_S has the highest ΔQ Mulliken value among the considered molecules. The ΔQ Mulliken values represent the charge transfer between the adsorbate molecule and the PdO-G composite surface, calculated using the Mulliken charge analysis method. The observed increase in ΔQ Mulliken values suggests a higher degree of charge transfer from the PdO-G surface to the adsorbate molecules as we move from NO_2_ to H_2_S. This phenomenon may be attributed to differences in the electronic structure, polarizability, and chemical bonding characteristics of the adsorbate molecules.

**Figure 6:**
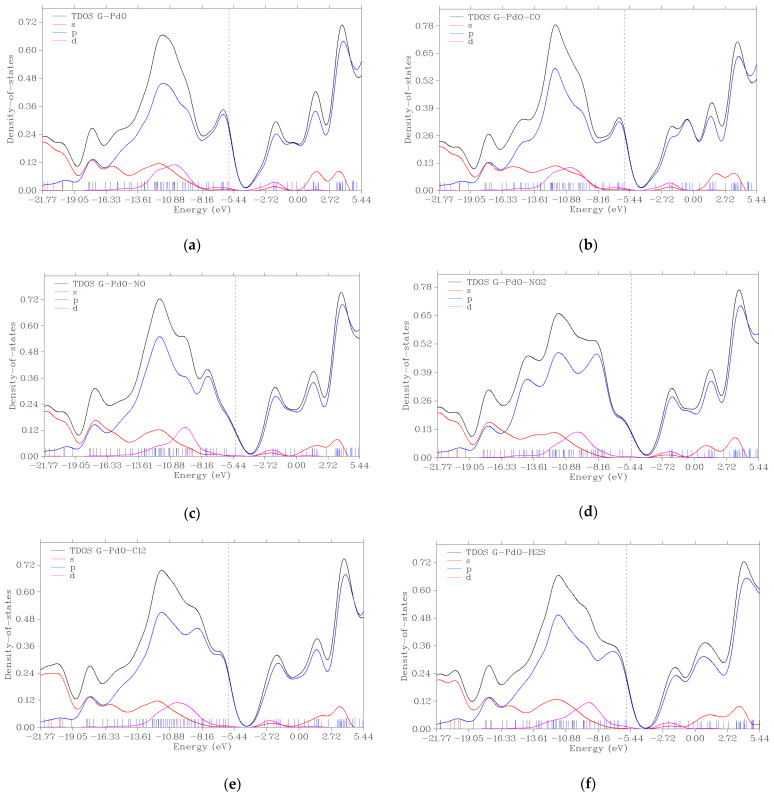
TDOS graphs of (**a**) PdO-G after optimization and after adsorption of (**b**) CO, (**c**) NO, (**d**) NO_2_, (**e**) Cl_2_, and (**f**) H_2_S, respectively.

To gain further insight into the nature of charge transfer within graphene composites, the TDOS of s, p, and d angular moment orbitals were obtained. As illustrated in Figure 6, near the Fermi level, only the peak of the *p*-orbital is observed. This observation is attributed to the sp^2^ hybridization π electron cloud, as reported by Kumar et al. [37]. Upon the formation of the composite, the peaks are slightly shifted towards the negative energy level. Additionally, more peaks emerge within the *p*-orbital, with greater peak spikes observed in the following order: G-PdO-CO, G-PdO-NO, G-PdO-Cl_2_, G-PdO-H_2_S, and G-PdO-NO_2_. These changes in the TDOS of the angular moment orbitals suggest alterations in the electronic structure of the composite upon the adsorption of the various gas molecules. The shifts and emergence of peaks indicate modifications in the density of electronic states, reflecting the interaction between the adsorbate molecules and the graphene composite.

The sensing performance of gas sensors is contingent upon several key parameters, including sensitivity, response time, selectivity, recovery time, etc. Improving sensing performance necessitates achieving considerable adsorption energy with the targeted gas molecule, as well as ensuring sufficient charge transfer between the gas and the sensor structure. These parameters play crucial roles in facilitating the spontaneous adsorption of the target gas molecule and influencing the electrical conductivity of the sensor. The electrical conductivity (*σ*) of the sensor is affected by the adsorption of gas molecules onto its surface [45,46].

(4) $$\sigma =AT^{3/2}\exp\left(\frac{-E_{g}}{2K_{b}T}\right)$$

where E_g_ represents the HOMO−LUMO energy gap, A is a constant (electrons/(m^3^ K^3/2^)), K_B_ is the Boltzmann constant (8.62 × 10^−5^ eV K^−1^), and T is the operating temperature in Kelvin. Adsorption events can induce changes in the electronic structure and charge distribution within the sensor material, leading to alterations in its conductivity. A sensor with a high sensitivity typically exhibits a significant change in conductivity upon exposure to the target gas, allowing for precise detection. In addition, achieving a rapid response time and recovery time is essential for real-time monitoring and efficient operation of the gas sensor. On the other hand, selectivity ensures that the sensor can distinguish the target gas from other gases present in the environment, thereby minimizing false alarms.

Based on the empirical equation, the derived sensitivity of the sensor is expressed as follows [47,48,49]:

(5) $$\mathrm{Sensitivity}\;(S)=\frac{\left|R_{2}-R_{1}\right|}{R_{1}}$$

where R_1_ and R_2_ are the electrical resistances of the composite before and after the adsorption process, respectively. The sensor’s sensitivity is expressed below after using the inverse relationship between electrical resistance and conductivity. The inverse relationship between electrical resistance and conductivity arises from their intrinsic material properties. According to Ohm’s law, electrical resistance is proportional to the resistivity of the material. Since resistivity is the reciprocal of conductivity, electrical resistance becomes inversely proportional to the conductivity of the material.

(6) $$S\;(\%)=\frac{\left|\sigma -\sigma_{0}\right|}{\sigma_{0}}\;=\exp\left(\frac{{\Delta E}_{g}}{K_{b}T}\right)-1$$

where *σ* and *σ*_0_ are the electrical conductivity of the gas sensor with and without the gas molecules, respectively. ${\Delta E}_{g}$ is the difference between the energy gap before and after the gas adsorption on the composite.

The strength of the gas adsorption is critically essential for measuring the desorption process. Higher adsorption strength hinders the process of desorption and leads to an increase in the sensor recovery time. Based on the transition states, the recovery time (τ) is given as follows [50]:

(7) $$\mathrm{Recovery}\;\mathrm{time}\;(\tau )=\nu^{-1}\exp\left(\frac{E_{\mathrm{ads}}}{K_{b}T}\right)$$

where *ν*, $K_{b}$, and T are the attempt frequency, Boltzmann constant, and operating temperature, respectively. $E_{\mathrm{ads}}$ is the adsorption energy of the gas molecule.

The Boltzmann constant is 8.62 × 10^−5^ eV K^−1^, and T is the operating temperature in Kelvin.

The attempt frequency and temperature are assumed to be kept constant with an order of ν = 1 THz at 298.15 K [51].

**Figure 7:**
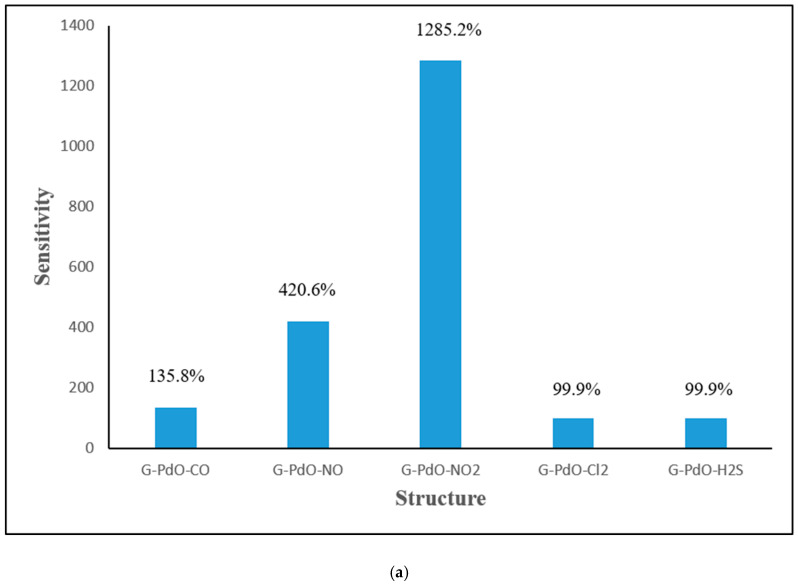

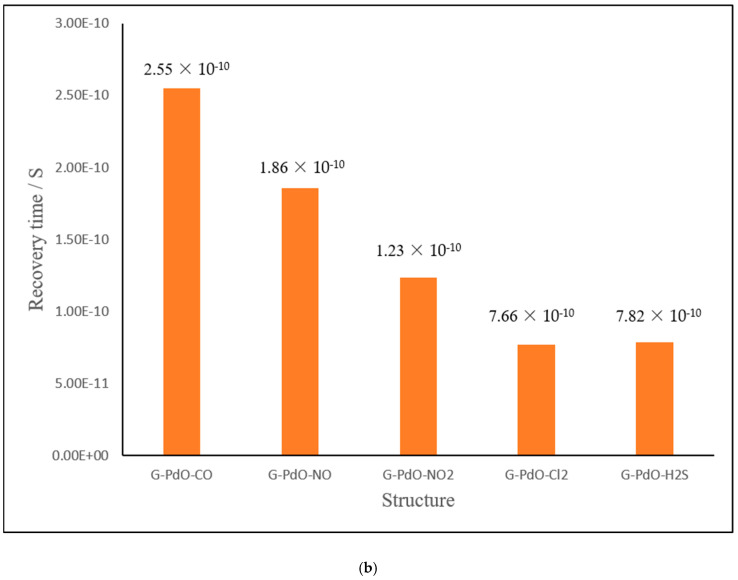
Calculated (**a**) sensitivity and (**b**) recovery time PdO-G after adsorption of CO, NO, NO_2_, Cl_2_, and H_2_S, respectively.

In Figure 7, the calculated sensing parameters of the sensitivity and recovery time at a constant temperature of 300 K are plotted. The analysis reveals that Cl_2_ gas exhibits the least sensitivity to PdO-G compared to the other gases. This reduced sensitivity can be attributed to the weaker reactivity and physisorption interaction of Cl_2_ gas with the PdO-G composite. The weaker reactivity and physisorption interaction of Cl_2_ gas with the PdO-G composite result in less pronounced changes in the sensor’s electrical conductivity upon exposure to Cl_2_ gas. Consequently, the sensor’s response to Cl_2_ gas is less sensitive, making it less effective in detecting Cl_2_ compared to the other gases. In general, the sensitivity of PdO-G composites tends to decrease as the magnitude of the charge transfer increases. This trend arises from the interplay between the charge transfer and the sensitivity of the sensor to the target gas molecules. A higher degree of charge transfer between the gas molecules and the PdO-G composite can lead to stronger adsorption interactions and more significant modifications in the electronic structure of the composite material. While such changes may enhance the overall reactivity of the composite towards gas molecules, they can also reduce the sensitivity of the sensor. An excessive charge transfer can saturate the available active sites on the sensor surface, limiting further adsorption of gas molecules and causing a decrease in sensitivity. Additionally, strong charge transfer may lead to changes in the conductivity of the sensor material, making it less responsive to subtle variations induced by the presence of target gas molecules. It was observed that the PdO-G composite exhibits greater sensitivity to the NO_2_ gas molecule compared to other gases. This heightened sensitivity may be attributed to several factors. NO_2_ is known to be highly reactive, and its interaction with the PdO-G composite may lead to significant changes in the electronic structure and conductivity of the composite material. NO_2_ molecules can form strong chemical bonds with the PdO-G composite surface, leading to efficient adsorption. The strong adsorption interaction facilitates the sensor’s detection of NO_2_ gas. NO_2_ adsorption on the PdO-G composite may involve substantial charge transfer between the gas molecule and the composite material. This charge transfer can induce noticeable changes in the electrical conductivity of the sensor, enhancing its sensitivity to NO_2_.

As indicated in Figure 7, the highest value of the recovery time was observed when CO is adsorbed on the PdO-G surface. This finding suggests that CO exhibits strong adsorption stability with graphene, which inhibits the desorption process from occurring efficiently. The prolonged recovery time associated with CO adsorption implies that CO molecules strongly adhere to the PdO-G surface, forming stable adsorption configurations. As a result, the desorption of CO from the surface is hindered, leading to a slower recovery process compared to other gas molecules. The strong adsorption stability of CO with graphene may arise from various factors, including chemical bonding interactions, surface morphology, and electronic properties of the PdO-G composite. These factors contribute to the formation of robust adsorption sites for CO molecules on the surface, enhancing their retention and delaying their release during the recovery phase. The decreasing order of the recovery time for the PdO-G composite with the different gas molecules is as follows: PdO-G-Cl_2_, PdO-G-H_2_S, PdO-G-NO_2_, PdO-G-NO, and PdO-G-CO. This order indicates that Cl_2_ exhibits the shortest recovery time, followed by H_2_S, NO_2_, NO, and CO. A shorter recovery time implies that the gas molecules desorb more rapidly from the PdO-G composite surface, allowing the sensor to return to its initial state more quickly after exposure to the gas.

The observed order of the recovery time may be influenced by factors such as the strength of the adsorption interactions between the gas molecules and the composite surface, the kinetics of desorption processes, and the surface properties of the PdO-G composite. In this context, Cl_2_ exhibits the shortest recovery time, suggesting weaker adsorption interactions or more rapid desorption kinetics compared to the other gases. Conversely, CO exhibits the longest recovery time, indicating stronger adsorption stability and slower desorption kinetics.

In accordance with the findings from the study by Qu Y. et al. [33], the properties of Pd-G were assessed for their suitability as sensing materials for CO and H_2_S gas molecules. Going further, according to our study, the properties of the PdO-G composite exhibit notably higher adsorption energies and band gaps for both CO and H_2_S gases compared to Pd-G. Specifically, the PdO-G composite demonstrates an adsorption energy of −6.5513 eV and a band gap of 2.5905 eV for CO gas, while for H_2_S gas, it displays an adsorption energy of −2.0110 eV and a band gap of 3.1321 eV. Conversely, Pd-decorated graphene exhibits comparatively lower adsorption energies and band gaps for both gases compared to PdO-G. This suggests that the PdO-G composite holds promise as a more effective sensing material for CO and H_2_S gas molecules, attributed to its stronger interactions and enhanced sensitivity, as indicated by the higher adsorption energies and band gaps observed. In accordance with the investigation conducted by Ma L. et al. [21], the efficacy of Pd-doped graphene as a sensing material for NO_2_ gas molecules was examined. The PdO-G composite demonstrates notably superior characteristics compared to Pd-G, according to the study conducted by Ma L. et al. Specifically, the composite exhibits a substantially higher adsorption energy (E_ad_ = −3.1665 eV) and a significantly shorter adsorption distance (d = 1.345 Å) for NO_2_ gas molecules, in contrast to the adsorption energy of 0.24 eV and adsorption bond length of 3.18 Å observed for Pd-G. These findings underscore the heightened affinity and closer proximity between the PdO–graphene composite and NO_2_ gas molecules. Consequently, the results suggest that the PdO-G composite is a more promising candidate for NO_2_ gas sensing applications compared to Pd-G.

According to the research findings presented by Shukri M. S. M. et al. [52], the adsorption characteristics of NO gas on Pd-doped graphene were investigated, revealing an adsorption energy of 3.916 eV and a Mulliken charge of −0.023 e. In our study, focusing on the PdO-G composite, we observed an enhanced adsorption energy of −4.7706 eV and a more negative Mulliken charge of −0.096 e for NO gas. These results suggest a more favorable interaction between the PdO-G composite and NO gas molecules compared to Pd-doped graphene. The higher adsorption energy and increased charge transfer observed in the composite indicate its potential superiority as a NO-gas-sensing material.

Finally, the results obtained from the previously conducted studies as in Table 2 [21,33] show that the PdO-G composite is more efficient than Pd-G and PG as a sensing material for CO, NO, NO_2_, and H_2_S.

**Figure 8:**
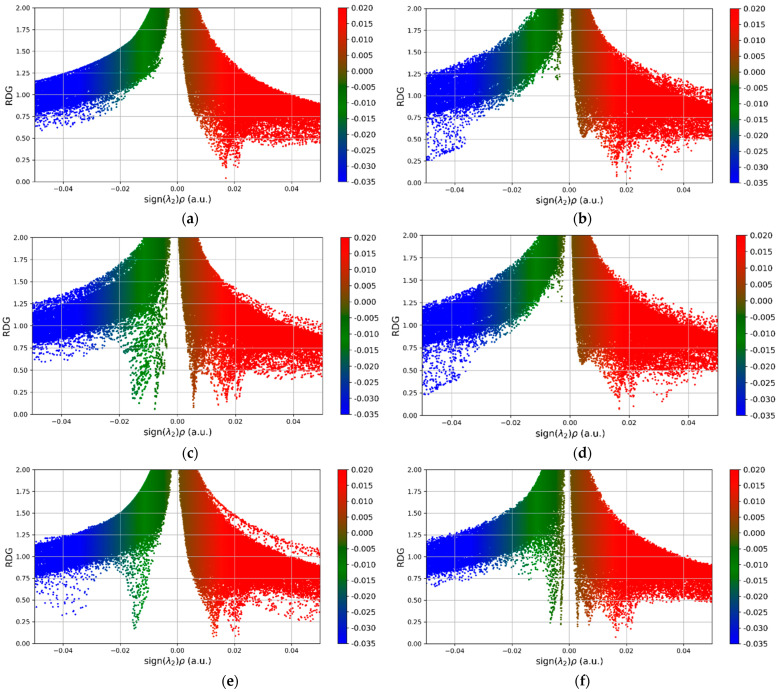
NCI graphs of (**a**) PdO-G after optimization and after adsorption of (**b**) CO, (**c**) NO, (**d**) NO_2_, (**e**) Cl_2_, and (**f**) H_2_S respectively.

Through observable changes in non-covalent interactions, Figure 8’s non-covalent interaction (NCI) plots for graphene sheets doped with PdO provide important insights into their sensitivity towards different gases, including NO_2_, CO, NO, H_2_S, and Cl_2_. For instance, upon NO_2_ adsorption, the PdO-G exhibits a highly responsive electrical environment, with the formation of new, strong attraction regions (blue) and strong repulsion regions (red). These alterations indicate the emergence of certain bonding interactions, including hydrogen bonding or π-π stacking, which point to a sensitive and dynamic interaction environment. The noticeable changes in the electron density (ρ) and the Laplacian of the electron density (∇^2^ρ) indicate that the PdO-G has a marked shift in interaction patterns, which indicates that it is a good fit for applications involving NO_2_ sensing.

The CO adsorption, on the other hand, has a more limited range of electron density values with significant positive Laplacian regions, indicating strong repulsive interactions and noticeable electron depletion. Higher CO concentrations cause the sensor to quickly become saturated, which reduces the sensor’s dynamic range and performance. Similar restrictions are shown in the NO adsorption, where repulsive interactions predominate and electron depletion zones are present. This emphasizes the need to balance repulsive and attractive interactions for optimal sensor performance. Furthermore, distinct properties of the interactions are revealed by comparing the electrical surroundings of various gases. Higher concentrations of data points in the strong attraction region of the H_2_S adsorption are suggestive of common hydrogen or halogen bonding interactions. The Cl_2_ adsorption exhibits a more uniform presence of strong interactions, likely due to halogen–halogen bonds and consistent van der Waals interactions.

The unique chemical and structural characteristics of each gas are highlighted by these observations, along with the consequences for sensor applications. In conclusion, PdO-G exhibits increased sensitivity and the potential for efficient gas detection, making it especially suited for NO_2_ sensing applications due to its broad range of electron density values and strong attractive contact formation.

Finally, the results obtained from previously conducted studies [21,33] show that the PdO–graphene composite is more efficient than Pd-G and PG as a sensing material for CO, NO, NO_2_, and H_2_S.

## 4. Conclusions

In this study, a comprehensive DFT investigation was conducted at the ground-state B3LYP level with LANL2DZ-labeled basis sets to explore the performance and design of a graphene-based PdO-G composite for detecting gases such as CO, NO, NO_2_, Cl_2_, and H_2_S. The structural, electrical, and adsorption characteristics of the PdO-G composite were studied, highlighting significant enhancements in sensor activity, gas sensitivity, selectivity, electronic structure, charge transfer, adsorption energies, interaction, structural optimization, and distortion.

The presence of Pd in the PdO-G composite causes significant distortions in the graphene structure, with optimized bond lengths averaging 1.964 Å (Pd-C) and 1.808 Å (Pd-O). Among the gases studied, CO exhibited the highest E_ads_ of −6.5513 eV, indicating strong interactions with the PdO-G composite, followed by NO, NO_2_, H_2_S, and Cl_2_, with Cl_2_ showing the weakest interaction.

Significant changes in the electronic structure, such as E_g_ and TDOS, were observed following gas adsorption. The charge transfer values further illustrate the differing levels of electron transfer associated with the adsorbed gases, with H_2_S showing the highest charge transfer of +0.445 e. The PdO-G composite exhibits strong chemical bonding and changes in electronic structure upon gas adsorption, making it particularly sensitive to NO_2_, while Cl_2_’s lower sensitivity can be attributed to its physical adsorption nature and lower reactivity.

The adsorption energy, besides the charge transfer occurring upon gas adsorption, affects the electrical conductivity of the sensors. Although excessive charge transfer can reduce the sensitivity by saturating active sites, higher adsorption energies typically correlate with increased sensitivities.

Overall, our findings demonstrate that PdO-G composites hold significant potential for enhancing the selectivity and sensitivity of graphene-based gas sensors. The strong interactions between PdO-G and gases, such as CO and NO_2_, suggest its viability for applications requiring high sensitivity. These results lay the foundation for developing advanced gas sensors with specific characteristics for detecting a variety of toxic gases, ultimately contributing to enhanced environmental safety and monitoring.

In conclusion, the PdO-G composite exhibited excellent gas detection performance, particularly for NO_2_, positioning it as a promising candidate for future sensor technologies.

## Figures and Tables

**Table 1:** Calculated adsorption energy (E_ads_), charge transfer of Mulliken (Q_Mulliken_), HOMO energy (E_HOMO_), LUMO energy (E_LUMO_), and HOMO−LUMO energy gap (E_g_) after adsorption of gas molecules.

	E_ads_ (eV)	Q_mulliken_ (e)	E_HOMO_ (eV)	E_LUMO_ (eV)	E_g_ (eV)
G-PdO-CO	−6.5513	0.036	−5.8640	−3.2735	2.5905
G-PdO-NO	−4.7706	−0.096	−5.2412	−2.6904	2.5508
G-PdO-NO_2_	−3.1665	−0.199	−5.3315	−2.8311	2.5004
G-PdO-Cl_2_	−1.9674	−0.070	−5.9566	−2.9222	3.0344
G-PdO-H_2_S	−2.0110	0.445	−5.8075	−2.6754	3.1321

**Table 2:** Comparison results of PdO-G, Pd-G, and PG for CO, NO, NO_2_, H_2_S, and Cl_2_ gas molecules.

Gas Molecule	PdO—G			PG			Pd—G		
	E_ads_ (eV)	ΔQ (e)	E_g_ (eV)	E_ads_ (eV)	ΔQ (e)	E_g_ (eV)	ΔQ (e)	E_g_ (eV)	E_g_ (eV)
CO	−6.5513	0.036	2.5905	0.08	0.015	-	1.05	0.155	0.023
NO	−4.7706	−0.096	2.558	−0.30	0.03	-	3.916	−0.023	-
NO_2_	−3.1665	−0.199	2.5004	0.24	0.204	-	2.17	0.663	-
H_2_S	−2.0110	0.445	3.1321	−0.36	-	0	1.285	0.242	0.071

## Data Availability

The original contributions presented in the study are included in the article, and further inquiries can be directed to the corresponding author.
